# Prolactin receptor signalling contributes to the attenuation of stress‐induced hyperthermia during pregnancy

**DOI:** 10.1111/jne.70261

**Published:** 2026-09-28

**Authors:** Andrew M. Stewart, David R. Grattan, Teodora R. Georgescu

**Affiliations:** ^1^ Centre for Neuroendocrinology and Department of Anatomy, Faculty of Biomedical Sciences University of Otago Dunedin New Zealand; ^2^ Maurice Wilkins Centre for Molecular Biodiscovery Auckland New Zealand

**Keywords:** lactogenic hormones, pregnancy, prolactin receptor, stress‐induced hyperthermia, thermoregulation

## Abstract

During pregnancy, thermoregulation is tightly controlled to protect the foetus from harmful elevated temperatures. Stress‐induced hyperthermia is a component of the stress response that increases alertness to a perceived threat. Previous literature has shown that like other aspects of the stress response, stress‐induced hyperthermia is attenuated in late pregnancy. We sought to investigate how stress‐induced hyperthermia is attenuated in pregnancy of mice. The lactogenic hormones, prolactin and placental lactogen, have been previously shown to act through the prolactin receptor (Prlr) to keep maternal temperature in pregnancy low to ensure successful reproductive outcomes. We hypothesised that Prlr signalling in the brain would attenuate stress‐induced hyperthermia during pregnancy. To investigate this, we specifically knocked out Prlr from glutamatergic (Vglut2) neurons in mice (*Prlr* ^lox/lox^/*Vglut2* ^Cre^). These neurons have been implicated in maternal adaptations and in stress‐induced hyperthermia. Using radiotelemetry to record core temperature (*T*_core_) and activity levels, non‐pregnant and pregnant mice were handled daily by scruffing the skin on the back of the neck to elicit a stress‐induced hyperthermic response. In pregnant control mice (*Prlr* ^lox/lox^), stress‐induced hyperthermia became attenuated from day 12 of pregnancy. This was not due to habituation to the stressor, as non‐pregnant mice did not show any attenuation of the stress‐induced *T*_core_ response following repeated handling for up to 20 days. In the *Prlr* ^lox/lox^/*Vglut2* ^Cre^ mice, the pregnancy‐induced attenuation of stress‐induced *T*_core_ was markedly delayed, only appearing at day 14 of pregnancy. The changes were specific to the temperature response to stress, as both genotypes displayed similar levels of stress‐induced activity following handling. Together, these data demonstrate that stress‐induced hyperthermia is attenuated during pregnancy in mice and that this effect is at least partially mediated by Prlr signalling in glutamate neurons. However, as the stress‐induced *T*_core_ response of the knockout mice was eventually attenuated in late pregnancy (gestation day 15–17), comparable to controls, other factors may also be involved.

## 1 INTRODUCTION

Thermoregulation is a highly regulated process controlled by the brain, allowing the body to adjust to and anticipate thermal challenges that pose a threat to homeostasis. Core temperature (*T*_core_) can fluctuate and increase due to a variety of reasons like sickness, physical activity and stress.^1^ During pregnancy, basal *T*_core_ increases due to the increased metabolic demands of the foetus.^2^ However, elevations in maternal temperature can be deleterious and are associated with an increased risk for maternal complications and damage to foetal growth and development.^3^, ^4^, ^5^, ^6^, ^7^, ^8^ To account for this, the mother employs a range of adaptations which minimise this risk by keeping *T*_core_ low. This includes suppressing circadian changes^2^ and fever responses^9^ through mechanisms like reducing brown adipose tissue (BAT) thermogenesis.^10^, ^11^ Stressful stimuli similarly challenge temperature homeostasis, which likely requires regulation during pregnancy.

The stress response is comprised of the fast catecholamine driven sympathetic–adreno–medullar (SAM) axis, followed by the slower corticosteroid‐driven hypothalamic–pituitary–adrenal (HPA) axis. Stress‐induced hyperthermia, a component of the SAM axis, arises from cortical signalling which activates neurons in the dorsomedial hypothalamus/dorsal hypothalamic area (DMH/DHA).^12^ This pathway projects to the rostral medullary raphe (rMR), which causes stress‐induced tachycardia, hypertension and metabolic shifts to fuel thermogenesis.^13^, ^14^ As stress‐induced hyperthermia occurs almost immediately following a stimulus, DMH/DHA neurons are thought to contribute to activation of corticotrophin‐releasing hormone (CRH) neurons in the paraventricular nucleus of the hypothalamus (PVH) neurons.^15^ This activates the HPA axis which elicits autonomic and behavioural responses, such as increases in adrenocorticotrophic hormone (ACTH) and subsequent cortisol (corticosterone in rodents) release from the adrenal glands, gluconeogenesis, lipolysis,^15^ locomotion^16^ and anxiety‐related behaviours.^17^ Stress‐induced hyperthermia therefore increases alertness to a perceived threat, allowing for a more robust response.

During pregnancy, the function of the SAM and HPA axes changes dramatically. Sympathetic tone increases during pregnancy, as evidenced by elevated sympathetic nerve activity,^18^ to enable required autonomic changes which help sustain pregnancy. This results in stressors, like inflammation, left unable to perturb the sympathetic nervous system responses.^11^ Similarly, while the basal levels of corticosterone increase throughout pregnancy, stress‐induced corticosterone release in late pregnancy and lactation is attenuated.^19^, ^20^ This is accompanied by reduced activity of the entire HPA axis.^21^ Consistent with pregnancy‐induced attenuation of SAM and HPA responses, there is some evidence that stress‐induced hyperthermia is altered in pregnancy. Rats exposed to an open field test show a robust increase in core body temperature. This temperature response was lost during pregnancy^22^ but follow‐up studies were unable to identify the mechanisms underlying this adaptation.^23^

There are multiple potential mechanisms underlying the pregnancy‐induced adaptations of the stress axes. Our focus has been on the role of the prolactin receptor (Prlr) in mediating adaptive changes in pregnancy. As well as mediating responses to the pituitary hormone, prolactin, Prlrs are also targeted by the pregnancy‐specific ligand, placental lactogen. The combined activation of Prlr by either or both hormones at different times throughout pregnancy and lactation makes this an attractive mechanism to mediate pregnancy and lactation‐specific adaptations. Canonically known for their role in mammary development and milk synthesis for lactation, the lactogenic hormones mediate a plethora of maternal adaptations including maternal behaviour,^24^, ^25^, ^26^, ^27^, ^28^ lactational infertility,^29^, ^30^ glucose homeostasis,^31^, ^32^ neurogenesis,^33^, ^34^ suppression of running wheel activity,^35^ temperature regulation^2^ and more. Pregnancy and lactation are characterised by reduced responsiveness of the stress axis,^21^ coinciding with increased signalling through the Prlr.^36^ Chronic exogenous prolactin treatment can dampen the HPA axis in non‐pregnant rats and reduces the associated anxious behaviour.^37^ Blocking the prolactin receptor (Prlr) exhibits the opposite, leading to increased anxiety behaviour and ACTH release.^38^ Prlr signalling may therefore also functionally dampen the SAM axis.

Prlr signalling modifies thermoregulatory mechanisms. Lactation is associated with reduced BAT thermogenesis due to low uncoupling protein 1 levels which can be replicated in hyperprolactinaemic rodent models.^39^ Prlr null mice undergo “beiging” of their white adipose, suggesting prolactin attenuates thermogenesis under normal conditions.^40^ Prolactin influences proper hair morphology development and sweating responses as evidenced in dairy cattle. Those with a loss of function mutation in the prolactin gene exhibit a higher *T*_core_, a hairier phenotype with reduced sweating and the inability to lactate, whereas a gain of function mutation in the Prlr gene confers resilience to heat.^41^ This aligned with previous research which observed elevations in prolactin levels coincided with reduced hair follicle growth in sheep.^42^ Hyperprolactinemia in male rats has also been shown to attenuate stress‐induced hyperthermia to restraint stress.^43^ Recently, our group discovered that Prlr signalling in glutamatergic (Vglut2) neurons is important to facilitate maintenance of a low maternal *T*_core_ during late pregnancy and to promote resilience to environmental increases in temperature.^2^ Vglut2 neurons in the dorsomedial hypothalamus/dorsal hypothalamic area (DMH/DHA) have also been shown to be crucial for stress‐induced hyperthermia signalling.^13^, ^14^ We therefore hypothesised that attenuation of stress‐induced hyperthermia in pregnancy would be mediated by Prlr signalling in glutamate neurons.

## 2 MATERIALS AND METHODS

### 2.1 Animals

*Vglut2*‐IRES‐Cre mice on a C57BL/6J background were purchased from the Jackson Laboratory (*Slc17a6* ^tm2(cre)Lowl^/MwarJ) and crossed with our *Prlr* ^lox/lox^ mice as detailed previously^2^, ^24^ to remove the Prlr from Vglut2 neurons (*Prlr* ^lox/lox^/*Vglut2* ^Cre^), with Cre‐negative mice serving as controls. There was an *n* = 6–10 per group for the pregnant experiment and *n* = 4–8 for the non‐pregnant experiment. Mice were housed in the Biomedical Research Facility at the University of Otago, Dunedin, New Zealand. Experimental procedures were approved by the University of Otago Animal Ethics Committee (Animal Use Protocol 22‐103) and carried out in accordance with the New Zealand Animal Welfare Act (1999).

Mice were individually housed in a controlled 12‐h light/dark cycle with lights on at 0600 h. The ambient temperature of the room was maintained at 30°C ± 1°C to reflect mice thermoneutral temperatures. As housing mice at lower ambient temperatures can act as a cold stressor, thermoneutrality was chosen to reduce sympathetic tone allowing us to isolate the effect of the stress on temperature. This also models human metabolism more effectively.^44^, ^45^ Mice had ad libitum access to food and water throughout experimentation.

### 2.2 Handling protocol

Mice were exposed to a minor handling stress between 0800 and 1000 h, which involved restraining them by the scruff of skin on their back for ~20 s. Handling began a minimum of 5 days after the surgical implantation of telemetry devices for monitoring *T*_core_ and activity levels (see below). Mice used for the non‐pregnant experiment were handled consecutively for 20 days regardless of oestrous cycle stage. Mice used in the pregnant experiment were handled for 3 days when non‐pregnant before a stud C57BL/6J mouse was placed in the cage for mating. Pregnancy was confirmed by presence of a copulatory plug and subsequent weight gain of ~1 g/day. Males were removed from the cage once pregnancy was confirmed.

### 2.3 Temperature and activity level recording and analysis

All mice had *T*_core_ and activity levels measured using a telemetry device (G2 E‐mitter implant, Starr Life Sciences Corp, USA). Prior to surgery, probes were sterilised and disinfected following the manufacturer's instructions. The device was surgically implanted into the peritoneal cavity of the animal and was adhered to the body wall muscle. The animal cages were then placed on top of ER4000 Energizer/Receiver bases (Starr Life Sciences Corp, USA) to begin recordings and data were collected and processed using the VitalView software program. Data were recorded at 0.0333 Hz and further averaged into 5‐min time bins using excel for analysis. Temperature data are represented as change from a 2 h baseline prior to handling. To determine if stress‐induced hyperthermia was attenuated in pregnancy and to account for stress‐induced hyperthermia amplitude and length, maximum area under the curve (AUC) from *t* = 0–120 min was used.

### 2.4 Statistical analyses

Statistical analyses were completed in GraphPad Prism (version 11.1.0). Analyses include investigating effects of genotype (G), gestation day (GD), time (T), handling day (HD) and their interactions. All analyses carried out were repeated‐measures two‐way analysis of variances (ANOVA) or mixed‐effect analyses if values were missing. Tukey's post hoc analysis was used when relevant. *p*‐value of <.05 was considered significant.

## 3 RESULTS

### 3.1 Stress‐induced hyperthermia is attenuated in pregnancy

To investigate if stress‐induced hyperthermia is altered in pregnancy and whether the lactogenic hormones are involved, we handled *Prlr* ^lox/lox^ (control) and *Prlr* ^lox/lox^/*Vglut2* ^Cre^ mice across pregnancy and measured changes in *T*_core_ in response to handling stress. For the initial analysis, handling responses were averaged across 3 days. During pregnancy, we observed a significant difference in the Δ*T*_core_ response to handling between the *Prlr* ^lox/lox^ and *Prlr* ^lox/lox^/*Vglut2* ^Cre^ mice at GD12‐14 (*F*_(1, 18)_ = 16.99, *p* = .0006, repeated‐measures two‐way ANOVA; Figure 1A) and a trend at GD15‐17 (*F*_(1, 18)_ = 5.615, *p* = .0292, repeated‐measures two‐way ANOVA; Figure 1A). At these timepoints, *Prlr* ^lox/lox^/*Vglut2* ^Cre^ mice had an elevated stress‐induced Δ*T*_core_ compared with the control mice.

**FIGURE 1:**
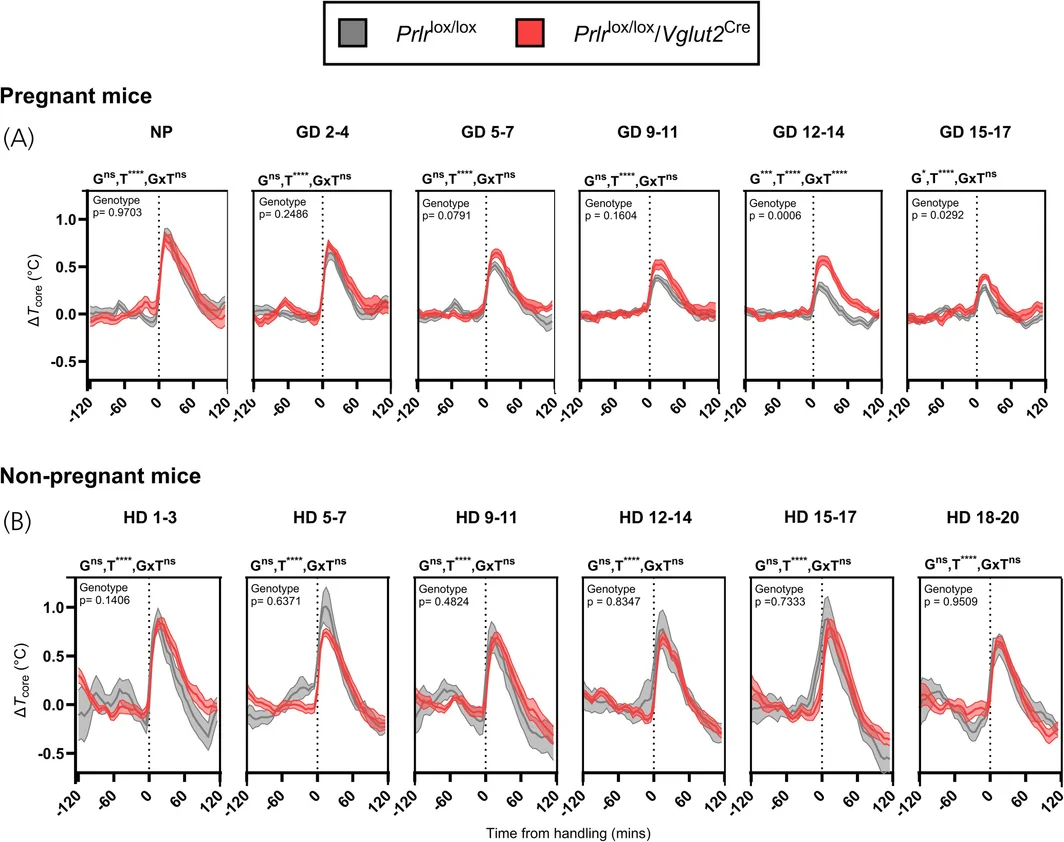
Stress‐induced hyperthermia is reduced in pregnancy. *Prlr* ^lox/lox^ pregnant mice show a progressive reduction in stress‐induced hyperthermia across pregnancy that was particularly evident by mid pregnancy at gestation day (GD) 12‐14. This attenuation of stress‐induced hyperthermia was significantly delayed in *Prlr* ^lox/lox^/*Vglut2* ^Cre^ mice with elevated Δ*T*_core_ at GD12‐14 and GD15‐17 (repeated‐measures two‐way ANOVA) (A). Non‐pregnant *Prlr* ^lox/lox^ and *Prlr* ^lox/lox^/*Vglut2* ^Cre^ mice exhibit no reduction in their stress‐induced hyperthermia following repeated handling for 20 days (repeated‐measures two‐way ANOVA) (B). Pregnant *Prlr* ^lox/lox^ *n* = 7–10, pregnant *Prlr* ^lox/lox^/*Vglut2* ^Cre^ *n* = 8–10, non‐pregnant *Prlr* ^lox/lox^ *n* = 4, non‐pregnant *Prlr* ^lox/lox^/*Vglut2* ^Cre^ *n* = 8. All data represented as mean ± SEM. Statistically significant effects for time (T), genotype (G) and their interaction are indicated by asterisks ns = not significant, *p < .05; **p < .01; ***p < .001 and ****p < .0001.

To investigate if amplitude and length of stress‐induced hyperthermia were impacted across time we analysed the data as AUC from *t* = 0–120 min after the handling (Figure 2). *Prlr* ^lox/lox^ control mice had a significantly reduced Δ*T*_core_ in response to handling at GD12‐14 and GD15‐17 compared to their non‐pregnant (NP) state and GD2‐4 (Effect of GD *F*_(3.819, 63.39)_ = 9.365, *p* < .0001, mixed effect analysis Tukey's post hoc analysis NP vs. GD12‐14 *p* = .0056, NP vs. GD15‐17 *p* = .0259; GD2‐4 vs. GD12‐14 *p* = .0045, GD2‐4 vs. GD15‐17 *p* = .0105; Figure 2A). This attenuation of the stress‐induced hyperthermia was not detected at any time during pregnancy in *Prlr* ^lox/lox^/*Vglut2* ^Cre^ mice. As matings were not timed based on the presence of proestrus, some of the pregnant mice were handled for longer than others. However, there was no difference in the maximum Δ*T*_core_ between those that received no extra handling and those which had up to 20 days of extra handling on GD‐1 or GD1 (Interaction days of extra handling × day of handling = *F*_(3, 6)_ = 1.447, *p* = .3197; Figure S1, Supporting Information).

**FIGURE 2:**
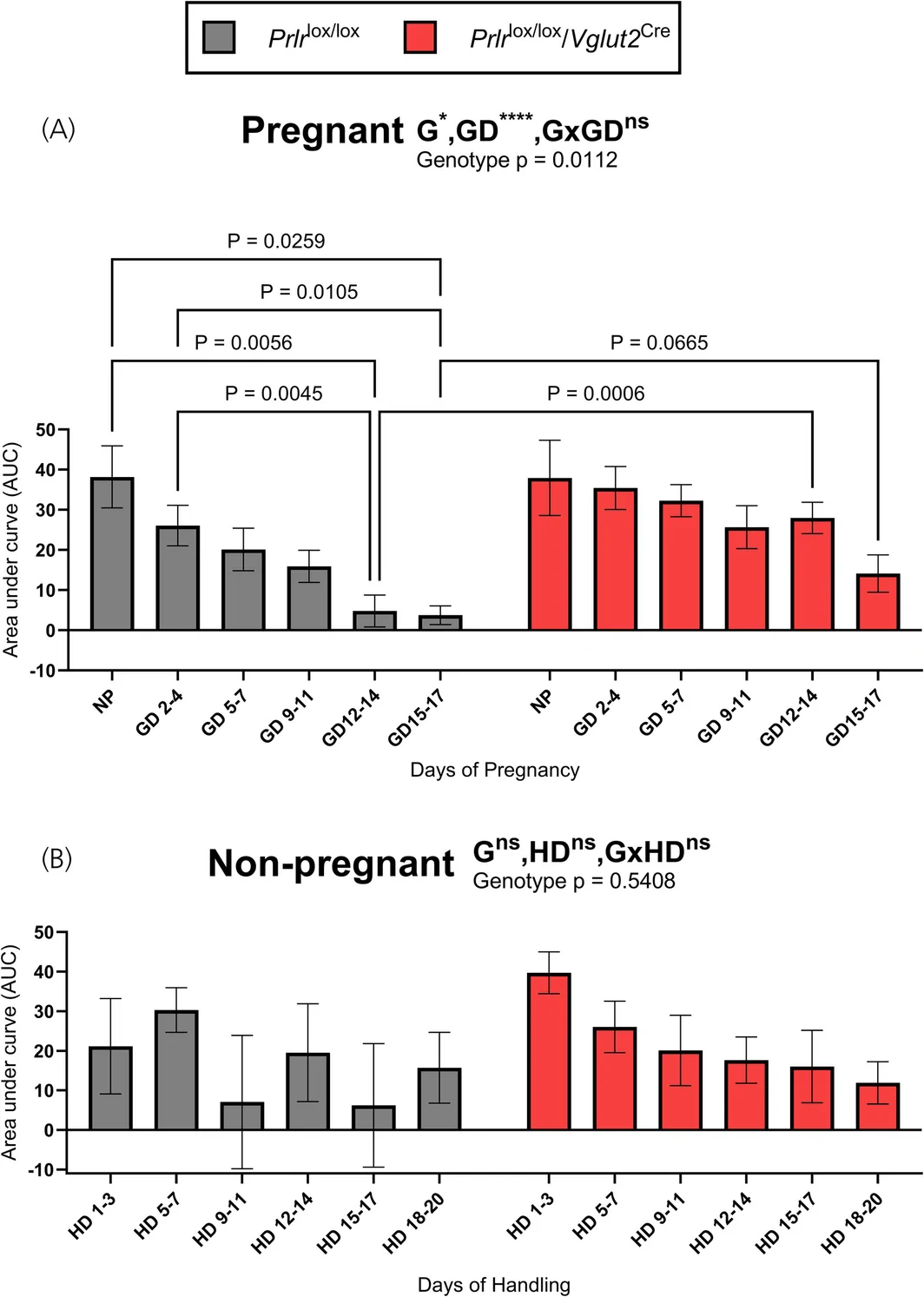
Lactogenic hormones contribute to the attenuation of stress‐induced hyperthermia. Area under the curve (AUC) analysis of data in Figure 1 shows that the stress‐induced temperature response was significantly reduced in control (*Prlr* ^lox/lox^) mice at GD12‐14 and GD15‐17 compared to non‐pregnant (NP) (mixed effect analysis followed by Tukey's post hoc analysis) (A). This response was markedly delayed in the Prlr knockout mice, such that the magnitude of the stress‐induced hyperthermia was significantly greater on GD12‐14. There was no difference in non‐pregnant responses after repeated handling for over 20 days (repeated‐measures two‐way ANOVA with Tukey's post hoc analysis) (B). Pregnant *Prlr* ^lox/lox^ *n* = 7–10, pregnant *Prlr* ^lox/lox^/*Vglut2* ^Cre^ *n* = 8–10, non‐pregnant *Prlr* ^lox/lox^ *n* = 4, non‐pregnant *Prlr* ^lox/lox^/*Vglut2* ^Cre^ *n* = 8. All data represented as mean ± SEM. Statistically significant effects for genotype (G), gestation day (GD), handling day (HD) and their respective interactions are indicated by asterisks ns = not significant, *p < .05; **p < .01; ***p < .001 and ****p < .0001.

To ensure this was a pregnancy‐specific effect, non‐pregnant mice of both genotypes were repeatedly handled to match the pregnant mice. In contrast to the pregnant animals, there was no change in the magnitude of the stress‐induced Δ*T*_core_ response across 3 weeks of repeated daily handling for either genotype (Effect of genotype *F*_(1, 10)_ = 0.4010, *p* = .5408, effect of HD *F*_(3.645, 36.45)_ = 2.101, *p* = .1061, repeated‐measures two‐way ANOVA; Figure 2B).

### 3.2 Lactogenic hormones act in mid pregnancy to reduce stress‐induced hyperthermia

To more accurately determine the time course for when the lactogenic hormones may be impacting stress‐induced hyperthermia, individual days of handling were plotted and analysed from the second half of pregnancy. We observed that in *Prlr* ^lox/lox^ mice stress‐induced hyperthermia was attenuated from GD12, becoming statistically significant at GD14 compared to GD11 (Effect of GD *F*_(5.016, 85.90)_ = 2.388, *p* = .0443, mixed effect analysis, Tukey's post hoc analysis *Prlr* ^lox/lox^ GD11 vs. GD14 *p* = .0420; Figure 3J). Removing the Prlr from Vglut2 neurons had the greatest impact on Δ*T*_core_ at GD12 and 13 with a significantly delayed attenuated Δ*T*_core_ in *Prlr* ^lox/lox^/*Vglut2* ^Cre^ mice compared to controls (Effect of genotype *F*_(1, 18)_ = 11.13, *p* = .0037, mixed effect analysis, Tukey's post hoc analysis *Prlr* ^lox/lox^ vs. *Prlr* ^lox/lox^/*Vglut2* ^Cre^ on GD12 *p* = .0017, GD13 *p* = .0304; Figure 3J). Stress‐induced hyperthermia eventually became attenuated in the *Prlr* ^lox/lox^/*Vglut2* ^Cre^ animals at GD14, 15, 16 and 17, as there was either only a trend towards significance, or no longer a difference between genotypes on these days (Figure 3G–J).

**FIGURE 3:**
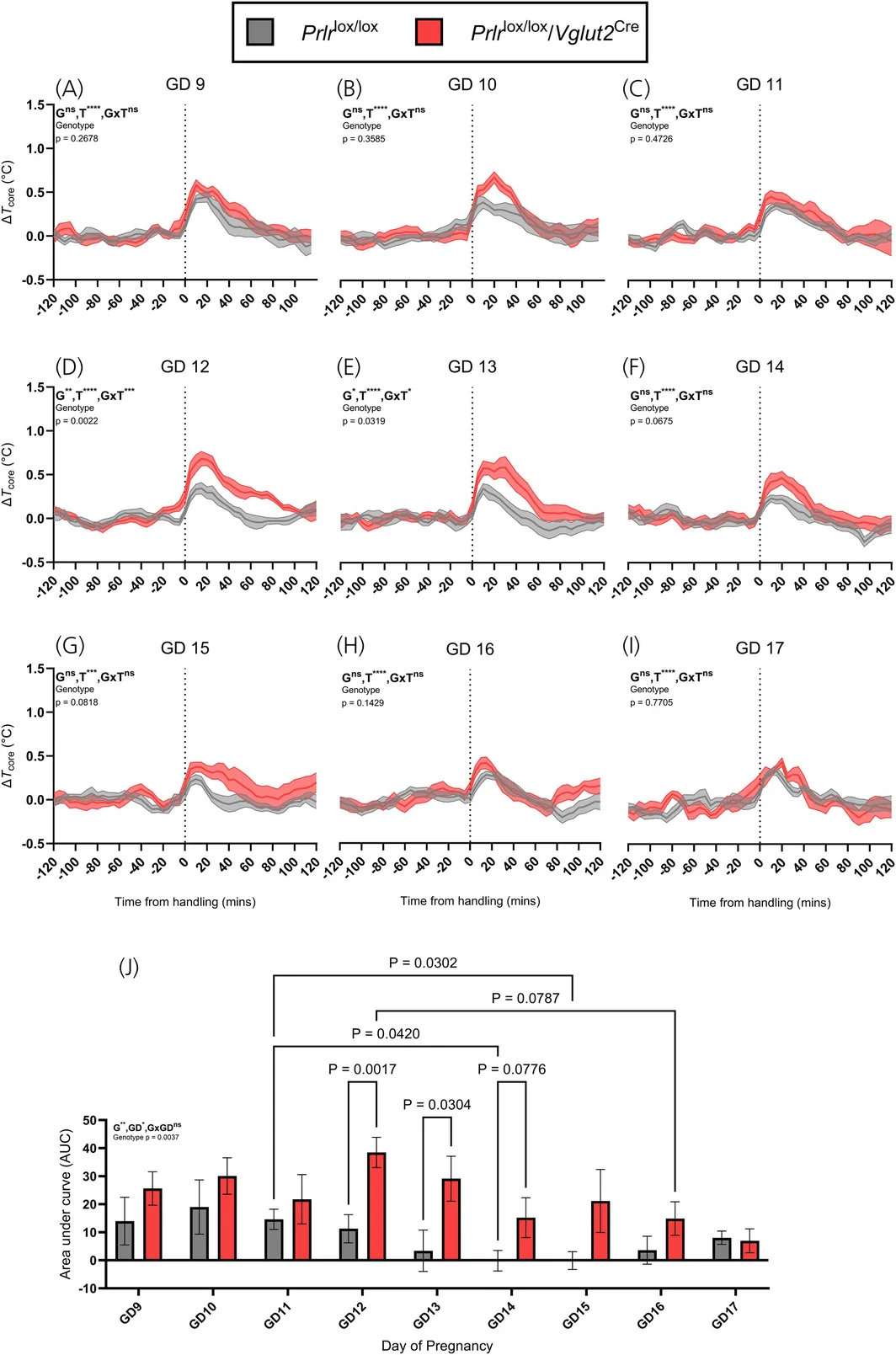
Lactogenic hormones act in mid pregnancy to reduce stress‐induced hyperthermia. *Prlr* ^lox/lox^/*Vglut2* ^Cre^ pregnant mice exhibit delayed attenuation of stress‐induced hyperthermia with significantly different Δ*T*_core_ at GD 12 and 13 (D, E and J) compared to *Prlr* ^lox/lox^ mice. There was no difference between groups on gestational day (GD) 9 (A), 10 (B), 11 (C), when responses were essentially similar to non‐pregnant animals. By day 14 (F), 15 (G), 16 (H) or 17 (I), there was again no significant difference between genotypes, when the attenuation of stress‐induced hyperthermia emerged in *Prlr* ^lox/lox^/*Vglut2* ^Cre^ animals to match the controls (repeated measures two‐way ANOVA, A–I; mixed effect analysis followed by Tukey's post hoc analysis, J). Pregnant *Prlr* ^lox/lox^ *n* = 9–10, pregnant *Prlr* ^lox/lox^/*Vglut2* ^Cre^ *n* = 6–10. All data represented as mean ± SEM. Statistically significant effects for time (T), genotype (G), gestation day (GD) and their respective interactions are indicated by asterisks ns = not significant, *p < .05; **p < .01; ***p < .001 and ****p < .0001.

### 3.3 Stress‐induced activity is not affected by Prlr signalling in pregnancy

Stressors can often cause changes in activity levels. In these same mice we measured activity to investigate if attenuation of stress‐induced hyperthermia in pregnancy was due to reduced activity following the handling. In the pregnant mice, we observed a main effect of pregnancy on activity (Effect of GD *F*_(3.927, 65.18)_ = 2.841, *p* = .0318, mixed effect analysis; Figure 4A,C), but not between genotype (Effect of genotype *F*_(1, 18)_ = 0.0467, *p* = .8313, mixed effect analysis; Figure 4A,C). Similarly, with the non‐pregnant mice, there was no difference between genotype at any timepoint (Effect of genotype *F*_(1, 10)_ = 0.1010, *p* = .7572, repeated‐measured two‐way ANOVA; Figure 4B,C). Curiously, in the non‐pregnant mice there was a significant effect of HD (Effect of HD *F*_(3.183, 31.83)_ = 3.807, *p* = .0177; which led to a reduction in stress‐induced activity for *Prlr* ^lox/lox^/*Vglut2* ^Cre^ mice at HD1‐3 to HD5‐7 and HD12‐14 (Effect of HD *F*_(3.183, 31.83)_ = 3.807, *p* = .0177, Tukey's post hoc analysis *Prlr* ^lox/lox^/*Vglut2* ^Cre^ HD1‐3 vs. HD5‐7 *p* = .0238, HD1‐3 vs. HD12‐14 *p* = .0227; Figure 4C)).

**FIGURE 4:**
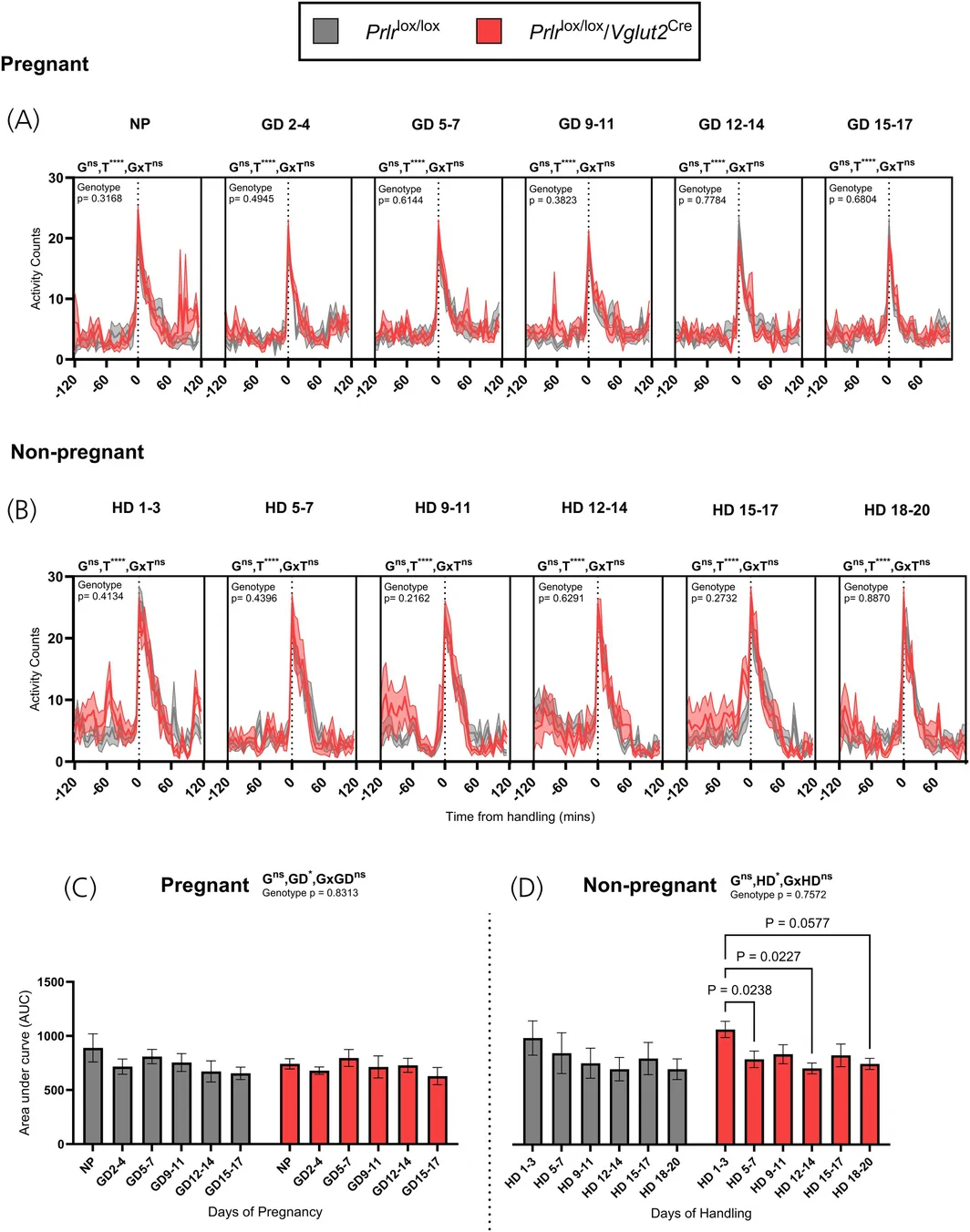
No genotype effect on activity following stress in pregnant mice. *Prlr* ^lox/lox^ and *Prlr* ^lox/lox^/*Vglut2* ^Cre^ pregnant mice (A, C) show no differences in activity between genotypes. Non‐pregnant mice show no difference between genotype (B) but exhibit some reductions across repeated handling days (HD) (D). Repeated measures two‐way ANOVA (A, B, D); mixed effect analysis (C) followed by Tukey's post hoc analysis, (C, D). Pregnant *Prlr* ^lox/lox^ *n* = 7–10, pregnant *Prlr* ^lox/lox^/*Vglut2* ^Cre^ *n* = 8–10, non‐pregnant *Prlr* ^lox/lox^ *n* = 4, non‐pregnant *Prlr* ^lox/lox^/*Vglut2* ^Cre^ *n* = 8. All data represented as mean ± SEM. Statistically significant effects for time (T), genotype (G), gestation day (GD), handling day (HD) and their respective interactions are indicated by asterisks ns = not significant, *p < .05; **p < .01; ***p < .001 and ****p < .0001.

## 4 DISCUSSION

These data demonstrate that stress‐induced hyperthermia is specifically attenuated in pregnancy. Our data support a role for Prlr signalling in contributing to this response through Prlr‐expressing Vglut2 neurons, which have been shown to have important roles in various aspects of thermoregulation, including stress‐induced hyperthermia.^13^, ^14^, ^46^, ^47^ Removing the Prlr from Vglut2 neurons resulted in delayed attenuation of the stress‐induced hyperthermic response, with the response in mid‐pregnant (GD12‐13) knockout animals remaining elevated compared to controls. However, from GD14‐17, the effect of the knockout was lost. This suggests a role for Prlr signalling on the attenuation of stress‐induced hyperthermia in mid pregnancy.

The specific ligand mediating actions through the Prlr has not been determined and is likely to differ at different timepoints across pregnancy. In early pregnancy (up to GD10), prolactin secreted from the maternal pituitary is likely the predominant ligand, as mating‐induced prolactin surges are critical to the establishment of pregnancy in rodents. From mid pregnancy, the placenta becomes the primary source of lactogenic hormones, with high placental lactogen concentrations increasing throughout the second half of pregnancy and acting through the Prlr.^48^ High placental lactogen results in the suppression of prolactin secretion from the maternal pituitary by activating the negative feedback pathways that normally regulate prolactin secretion. In late pregnancy, pituitary prolactin secretion is reactivated for a period leading up to parturition.^49^ Hence, for the major part of the observed response, it seems likely that the transition from prolactin to placental lactogen dominance (around GD10‐12) is the time of greatest impact of Prlr signalling on the stress response. In late pregnancy however, additional mechanisms may contribute to suppressing stress‐induced hyperthermia. One possibility is that pituitary prolactin may act on other neuronal populations other than Vglut2 neurons. Alternatively, other hormones like oestrogens, which increase in murine pregnancy from GD14^49^ and are involved in maternal adaptations^50^, ^51^, ^52^ and reducing temperature,^53^ could similarly be involved in attenuating stress‐induced hyperthermia.

One interesting observation that came from our work was that stress‐induced hyperthermia from handling did not habituate in non‐pregnant female mice, despite females being previously described to habituate better to stressors than males.^54^ This adds to the array of studies (summarised in Reference)^55^ reporting different results dependent on stressor type and context. However, most of these studies evaluated the stress‐induced hyperthermia occurring during the stressor, as opposed to the post‐stress effect as seen in the present study. Previous experimental protocols have also used types and bouts of stress that are more stressful than the short and mild stressor used in the current study. What is unique about our research, is that despite only being handled for 20 s, and behaviourally appearing to get used to this handling, the mice never physiologically habituated to this stressor and continued to display an equivalent increase in body temperature each time. One possibility is that larger stress responses may be required before habituation to the stressor is observed. This reinforces the concept that the attenuation is a specific temperature effect in pregnancy, as previous studies have reported reduced temperature in more stressful paradigms like an open field, yet elevated corticosterone responses.^56^

We were unable to control for oestrous cycle in the present study due to investigating the impact of repeated handling. The fluctuating levels of hormones during different oestrous cycle stages can impact thermoregulation,^57^ for example rat *T*_core_ increases during proestrus when progesterone levels are high.^58^, ^59^ Administration of progesterone or oestrogen to ovariectomised rats also increases and decreases temperature respectively. This could potentially account for some of the variability seen in the non‐pregnant Δ*T*_core_ data but would not account for the lack of habituation. These data further reinforce that the attenuation of stress‐induced hyperthermia is a pregnancy driven effect.

The data suggests that the attenuation of the stress‐induced hyperthermia was not associated with a genotype‐dependent reduction in stress‐induced activity in response to the handling. Our data showed that despite the progressive reduction in stress‐induced hyperthermia, this is accompanied by a subtle reduction in activity following handling throughout pregnancy. However, as there was no effect of genotype, this reduction in activity did not account for the attenuation of stress‐induced hyperthermia observed in the controls compared to the knockouts. It suggests this is not a global suppression of the stress response, but rather a specific action to prevent uncontrolled increases in *T*_core_ during pregnancy. Other thermoeffectors may therefore be altered following stress in pregnancy, as these have been considered the major drivers of stress‐induced hyperthermia.^60^, ^61^

Our data have shown that Prlr signalling on Vglut2 neurons is important for modulating stress‐induced hyperthermia in pregnancy. Vglut2 is expressed by multiple populations shown to be involved in stress‐induced hyperthermia; however, next steps should aim to determine the mechanism of action and location through which Prlr signalling is eliciting this effect. As we have previously shown, AAV‐mediated deletion of the Prlr from the POA leads to increased *T*_core_ throughout pregnancy.^2^ The effect of glutamate‐specific deletion of Prlr on stress‐induced hyperthermia was observed in a much more limited time window in mid pregnancy, peaking around GD12‐14. It seems likely that this represents a distinct mechanism from that regulating basal thermal homeostasis. Brain regions involved in stress‐induced hyperthermia, such as the DMH and rMR, may be involved as both regions express the Prlr,^62^ but this requires further investigation.

Several limitations of the present study should be considered when interpreting these findings. As matings were not timed, males remained in the dam's cage until pregnancy was confirmed (no later than GD12), resulting in some mice being handled for longer. However, this is unlikely to impact our results as mice that received no extra handling had no different maximum Δ*T*_core_ to those mice handled up to 20 days extra, reinforcing that the observed attenuation of stress‐induced hyperthermia is pregnancy‐induced and not due to habituation. All mice also shared similar baseline levels on days where significant genotype effects were observed, and AUC analyses only considered Δ*T*_core_ 0–120 min post handling, specifically highlighting the post‐stress response. Developmental compensation in the *Prlr* ^lox/lox^/*Vglut2* ^Cre^ mice must also be considered when discussing the role of Prlr signalling in thermoregulation. Finally, although it also remains unknown whether repeated exposure to a mild handling stressor can alter central Prlr expression, our work to date suggests that changes in Prlr signalling rely on changes in hormone secretion during pregnancy rather than a change in receptor expression itself, although we cannot exclude the latter.

Collectively, this study demonstrates another adaptive thermoregulatory response used in pregnancy to keep dams cool. This overrides a beneficial, conserved evolutionary response to stress to protect against a thermal challenge and negate adverse outcomes for the pregnancy.

## AUTHOR CONTRIBUTIONS

**David R. Grattan:** Conceptualization; formal analysis; writing – review and editing; funding acquisition; methodology; supervision. **Andrew M. Stewart:** Conceptualization; data curation; formal analysis; methodology; writing – review and editing; writing – original draft; investigation. **Teodora R. Georgescu:** Supervision; writing – review and editing; funding acquisition; data curation; conceptualization; methodology; formal analysis; investigation.

## CONFLICT OF INTEREST STATEMENT

The authors declare no conflicts of interest.

## ETHICS STATEMENT

All animal experimental protocols were approved by the University of Otago ethics committee.

## Supporting information


**Figure S1.** No difference is observed in stress‐induced hyperthermia between mated mice who underwent different lengths of handling prior to pregnancy on gestation day one or the day prior (*n* = 1–3 per group).

## Data Availability

The data that support the findings of this study are available from the corresponding author upon reasonable request.
